# Icariside II, a novel phosphodiesterase 5 inhibitor, protects against H_2_O_2_‐induced PC12 cells death by inhibiting mitochondria‐mediated autophagy

**DOI:** 10.1111/jcmm.12971

**Published:** 2016-09-19

**Authors:** Jianmei Gao, Yuanyuan Deng, Caixia Yin, Yuangui Liu, Wei Zhang, Jingshan Shi, Qihai Gong

**Affiliations:** ^1^ School of PharmacyZunyi Medical UniversityGuizhouChina; ^2^ Department of Pharmacology and Key Laboratory of Basic Pharmacology of Ministry of EducationZunyi Medical UniversityGuizhouChina

**Keywords:** icariside II, reactive oxygen species, autophagy, mitochondria, glycogen synthase kinase‐3β

## Abstract

Oxidative stress is a major cause of cellular injury in a variety of human diseases including neurodegenerative disorders. Thus, removal of excessive reactive oxygen species (ROS) or suppression of ROS generation may be effective in preventing oxidative stress‐induced cell death. This study was designed to investigate the effect of icariside II (ICS II), a novel phosphodiesterase 5 inhibitor, on hydrogen peroxide (H_2_O_2_)‐induced death of highly differentiated rat neuronal PC12 cells, and to further examine the underlying mechanisms. We found that ICS II pre‐treatment significantly abrogated H_2_O_2_‐induced PC12 cell death as demonstrated by the increase of the number of metabolically active cells and decrease of intracellular lactate dehydrogenase (LDH) release. Furthermore, ICS II inhibited H_2_O_2_‐induced cell death through attenuating intracellular ROS production, mitochondrial impairment, and activating glycogen synthase kinase‐3β (GSK‐3β) as demonstrated by reduced intracellular and mitochondrial ROS levels, restored mitochondrial membrane potential (MMP), decreased p‐tyr216‐GSK‐3β level and increased p‐ser9‐GSK‐3β level respectively. The GSK‐3β inhibitor SB216763 abrogated H_2_O_2_‐induced cell death. Moreover, ICS II significantly inhibited H_2_O_2_‐induced autophagy by the reducing autophagosomes number and the LC3‐II/LC3‐I ratio, down‐regulating Beclin‐1 expression, and up‐regulating p62/SQSTM1 and HSP60 expression. The autophagy inhibitor 3‐methyl adenine (3‐MA) blocked H_2_O_2_‐induced cell death. Altogether, this study demonstrated that ICS II may alleviate oxidative stress‐induced autophagy in PC12 cells, and the underlying mechanisms are related to its antioxidant activity functioning *via* ROS/GSK‐3β/mitochondrial signalling pathways.

## Introduction

Oxidative stress plays a key pathologic role in neurodegenerative diseases (considered as redox diseases) such as Alzheimer's disease and Parkinson's disease 1. Abnormal high levels of H_2_O_2_ in neurons have been reported in neurodegenerative diseases, which is suggested to be responsible for the oxidative stress‐induced neuronal damage 2. H_2_O_2_‐induced reactive oxygen species (ROS) function as important physiological regulators of intracelluar signalling pathways, and the dysregulation of ROS signalling may contribute to redox diseases 3. Remedies targeting the oxidative stress and the antioxidant response may be promising strategies for treating redox diseases. However, drug development is being hindered by limited understanding of pharmaceutically relevant molecular targets and mechanisms in most oxidative stress‐mediated redox diseases 4.

Accumulating evidence indicates that mitochondrial dysfunction plays a key role in the pathophysiology of many neurological diseases. Mitochondria are critical for meeting the high energy demands of the brain, while they also generate the majority of intracellular ROS, which can cause oxidative damage to important cellular structures and alteration of redox signalling 5. In mammalian cells, mitochondrial ROS production and oxidation of mitochondrial lipids seem to play a role in autophagy 6. Moreover, previous evidence suggests that GSK‐3β is implicated in modulating the oxidative status and in regulating autophagy 7. GSK‐3β is a serine/threonine kinase, the activation of which is indicated by phosphorylation at tyr216 or dephosphorylation at ser9 8. GSK‐3β is involved in redox diseases such as bipolar disorder, schizophrenia and Alzheimer's disease. Overexpression of GSK‐3β can induce autophagy in neurons, and GSK‐3β can modulate nuclear localization of the transcription factor Nrf2, which is now considered a master regulator of redox homeostasis and defence against ROS. Moreover, GSK‐3β can be activated by the antioxidant response 9. Therefore, when a cell is challenged by oxidative stress, the regulation of expression of GSK‐3β and anti‐autophagy mitochondrial signalling pathway are critical in determining the cell fate. However, the crosstalk between autophagy, redox signalling, and mitochondrial dysfunction remains unclear 10.

Phosphodiesterase 5 (PDE5) is a 3′,5′ cyclic guanosine monophosphate (cGMP)‐specific hydrolase, which has recently attracted much interest not only in erectile dysfunction but also in neurodegenerative diseases 11. PDE5 inhibitors regulate signalling pathways by elevating levels of cGMP, and pathways specific to cGMP have been characterized extensively, particularly since the discovery of nitric oxide as a neuroregulatory molecule. Indeed, nitric oxide activity is primarily mediated *via* increases in cellular cGMP levels. Thus, the main mechanism of action of PDE5 inhibitors such as sildenafil probably involves the increased levels of cGMP in cells within the central nervous system. Interestingly, association between nitric oxide/cGMP signalling and GSK‐3β activity is supported by the existence of a signalling cascade that links cGMP activation with GSK‐3β inhibition 12. Targeting PDE5 has recently gained much interest in several neurodegenerative diseases. For example, PDE5 inhibitors have been reported to be neuroprotective and improve cognitive and motor functions in various experimental neurodegenerative models, and have been reported to have therapeutic potential in redox diseases 13, 14.

**Figure 1:**
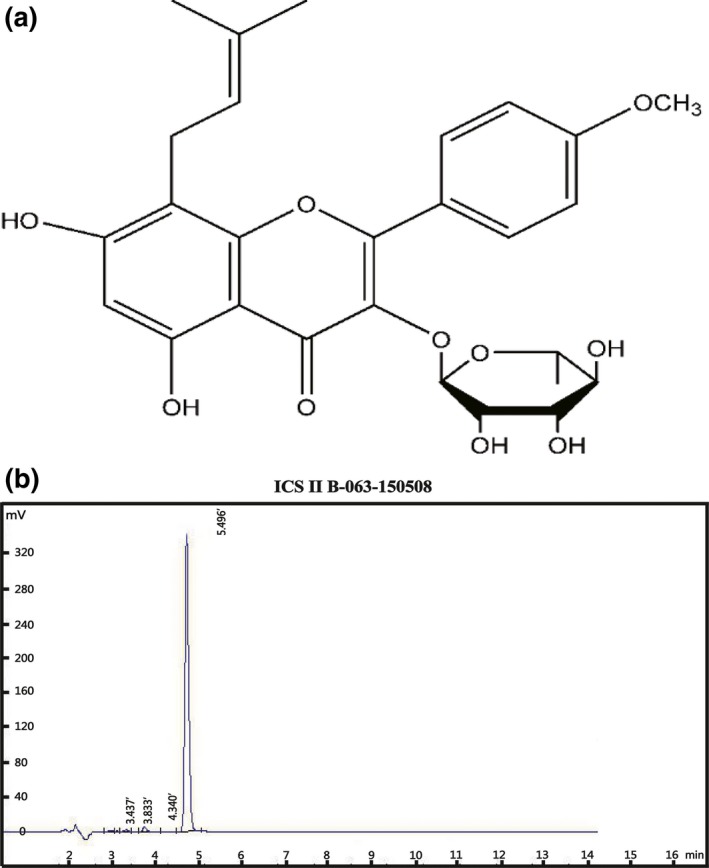
The chemical structure and high‐performance liquid chromatography of ICS II.

Chinese herbal medicines are rich resources to discover novel PDE5 inhibitors 15. Herbal *Epimedii* is a herbal medicine extensively used in China for the treatment of dementia, osteoporosis, cardiovascular diseases, and for immunomodulation function regulation 16. ICS II (Fig. 1) is considered to be one of the major pharmacologically active components of herbal *Epimedii*. Several studies have demonstrated that ICS II has antioxidant capacity and exerts neuroprotective effect such as the alleviation of cerebral ischaemia/reperfusion‐induced neuronal injury and streptozotocin‐induced cognitive deficits in rats 17, 18, 19, 20. However, whether ICS II has protective effect on the oxidative stress‐induced mitochondrial dysfunction and autophagy in neuron has not yet been studied.

This study was designed to investigate *in vitro* whether ICS II exerts a protective effect on rat neuronal PC12 cells against H_2_O_2_‐induced oxidative damage, and further explore the underlying molecular mechanism.

## Materials and methods

### Reagent

Icariside II (ICS II, purity ≥ 98%) was purchased from Nanjing Zelang Medical Technology Corporation Ltd. (Nanjing, China), and N‐acetyl‐L‐cysteine (NAC) was purchased from Sigma‐Aldrich (St Louis, MO, USA). Both compounds were dissolved in dimethyl sulfoxide (DMSO) at 10 mM as stock solution and diluted in culture medium respectively. The final concentration of DMSO in the media was less than 0.01%. H_2_O_2_ (H1009), 3‐(4,5‐dimethythiazol‐2‐yl)‐2,5‐diphenyltetrazo‐lium bromide (MTT)(M2128), 3‐MA (M9281), SB216763 (S3442), rhodamine 123 (R8004), Monodansylcadaverine (MDC) (30432), 2′,7′‐dichlorodihydrofluorescein diacetate (DCFH‐DA) (D6883) were purchased from Sigma‐Aldrich, lactate dehydrogenase (LDH) assay kit, nitric oxide, phosphodiesterase 4 (PDE4), PDE5, 3′,5′ cyclic adenosine monophosphate (cAMP) and cGMP ELISA kit were purchased from Shanghai Jiang Lai Biotechnology (Shanghai, China), MitoSOX Red (M36008) and Mito Tracker green probe **(**M7514) were purchased from Invitrogen (Eugene, OR, USA), anti‐LC3B (ab48394), anti‐Beclin 1 (ab55878), anti‐p62/SQSTM1 (ab56416), anti‐HSP60 (ab46798), anti‐GSK‐3β (ab93926), anti‐p‐ser9‐GSK‐3β (ab75814), anti‐p‐tyr216‐GSK‐3β (ab75745) were purchased from Abcam (Cambridge, UK).

### Cell culture and treatment

The highly differentiated rat pheochromocytoma line PC12, a clonal cell line derived from a rat adrenal medulla tumour, was obtained from American Type Culture Collection (Rockville, MD, USA). Cells were cultured in DMEM medium supplemented with 10% foetal bovine serum, 2 mM L‐glutamine, penicillin (100 U/ml) and streptomycin (100 μg/ml), and maintained at 37°C, 5% CO_2_ in a humidified atmosphere. The PC12 cells (2 × 10^4^ cells/well in 96‐well plates) at 37°C were pre‐treated with ICS II for 1 hr and thereafter exposed to 400 μM H_2_O_2_ for a further 48 hrs. The autophagy inhibitor 1 mM 3‐MA and 20 μM GSK‐3β inhibitor SB216763 were added to the particular cell cultures 1 hr before ICS II treatment respectively.

### Cell viability determination

Briefly, before the end of the treatments, 5 mg/ml MTT was added to each well for 4 hrs at 37°C. The dark‐blue formazan crystals formed in intact cells were dissolved in DMSO and their absorbance was measured at 490 nm with a microplate reader. Results were expressed as the percentage of MTT reduction relative to the absorbance of the control cells. In parallel experiments, NAC (20 μM) was used as a positive control for antioxidant activity that limits cell death, for comparison with the effect exerted by ICS II.

### Measurement of lactate dehydrogenase release

The level of LDH released by PC12 cells under various treatments (described above) was determined by using a LDH assay kit according to the manufacturer's instructions. In brief, PC12 cells were treated as described above and the supernatant was used in the assay. At the end of the experiment, supernatant was collected from each well and centrifuged at 400 g for 5 min. To determine the LDH activity in the supernatant, 100 μl of freshly diluted reaction mixture, consisting of catalyst and dye solution, were mixed with 50 μl of supernatant and plated in a 96‐well plate protected from light for 30 min. before absorbance was read. Absorbance was read at 490 nm. As a positive control, LDH contained in cells, were lysed in 1% Triton X‐100.

### Observation of morphologic changes

PC12 cells were treated as described above. After 48 hrs, cellular morphologic changes were observed using phase contrast microscopy.

### Measurement of intracellular ROS levels

The fluorescent probe DCFH‐DA was used to detect intracellular ROS. Briefly, PC12 cells were treated as described above for 48 hrs. After being washed with phosphate buffer saline (PBS), cells were labelled with 20 μM DCFH‐DA for 30 min. Fluorescence levels were measured using a fluorescence reader with excitation at 485 nm and emission at 530 nm (Varioskan Flash Multimode Reader).

### Mitochondrial ROS analysis

Mitochondrial ROS was evaluated using MitoSOX Red, a fluorescent indicator for mitochondrial superoxide 21. Briefly, PC12 cells were treated as described above, cells were washed with balanced salt solution (HBSS) and loaded with 5 μM MitoSOX Red at 37°C in the dark for 20 min. Thereafter, the cells were washed with PBS and incubated with 200 nM Mito Tracker green probe and kept at 37°C for 20 min. Subsequently, the cells were washed with PBS and imaged by fluorescence microscopy (Olympus IX73; Olympus, Tokyo, Japan; 200×) with excitation/emission (510/580 nm) filters.

### Determination of MMP

Mitochondrial membrane potential was determined as described in our previous study 22. In brief, PC12 cells were treated as described above. Thereafter, cells were stained with 2 μg/ml rhodamine 123 for 20 min. in the dark, and then were washed with PBS. Mitochondrial uptake of rhodamine 123 was observed by fluorescence microscopy (Olympus IX73; Olympus; 200×) with excitation/emission (485/595 nm) filters.

### Determination of nitric oxide, PDE4, PDE5, cAMP and cGMP level

In brief, PC12 cells were treated as described above. Cells were lysed in the lysis buffer and centrifuged for 20 min. at 12,000 × g at 4°C. The supernatant was stored at −80°C for subsequent measurement. The levels of nitric oxide, PDE4, PDE5, cAMP and cGMP were quantified using the nitric oxide, PDE4, PDE5, cAMP and cGMP ELISA kit according to the manufacturer's indications.

### Detection of autophagic vacuoles

MDC staining of autophagic vacuoles formation was performed for autophagy analysis 23. PC12 cells were pre‐treated with different concentration of ICS II for 1 hr before incubation with 400 μM H_2_O_2_. After 48 hrs, autophagic vacuoles were labelled with 0.05 mM MDC in PBS at 37°C for 30 min. After incubation, cells were washed three times with PBS and immediately analysed under by fluorescence microscopy (Olympus IX73, Olympus; 200×) with excitation/emission (380/530 nm) filters. The mean fluorescence intensity was measured by the Image Pro Plus software (Version 6.0, Media Cybernetics, LP, USA).

### Western blot analysis

PC12 cells were pre‐treated with different concentrations of ICS II for 1 hr before exposure to 400 μM H_2_O_2_. After 48 hrs, cells were lysed in the lysis buffer, and proteins were quantified using the BCA protein assay kit. Following electrophoresis, proteins were blotted onto a PVDF membrane. The membrane was blocked with 5% non‐fat milk at room temperature for 2 hrs and incubated overnight at 4°C with the appropriate primary antibody: anti‐LC3B (1:1000), anti‐Beclin 1 (1:1000), anti‐p62/SQSTM1 (1:1000), anti‐HSP60 (1:1000), anti‐GSK‐3β (1:1000), anti‐p‐ser9‐GSK‐3β (1:1000), anti‐p‐tyr216‐GSK‐3β (1:1000). Thereafter, the membranes were incubated with horseradish peroxidase‐conjugated secondary antibodies for 2 hrs at room temperature under shaking. Immunoreactive proteins were visualized with the ECL Western blot detection kit. The image was scanned and band optical intensity was quantified using Quantity One 1‐D analysis software v4.52 (BioRad, Philadelphia, USA).

### Statistical analysis

All results were analysed by the SPSS 16.0 statistics software and were presented as mean ± S.D. One‐way anova was used for multiple group comparisons. When anova test results for all data were significant, *post hoc* least significant difference (LSD) was used to determine individual differences. A value of *P* < 0.05 was considered significant. All results were confirmed in at least three independent experiments.

## Results

This study found that ICS II, a novel PDE5 inhibitor, exerted neuroprotective effect against H_2_O_2_‐induced autophagy by inhibition of the ROS/GSK‐3β/mitochondrial signalling pathways in rat neuronal PC12 cells.

### ICS II attenuated H_2_O_2_‐induced cytotoxicity in PC12 cells

**Figure 2:**
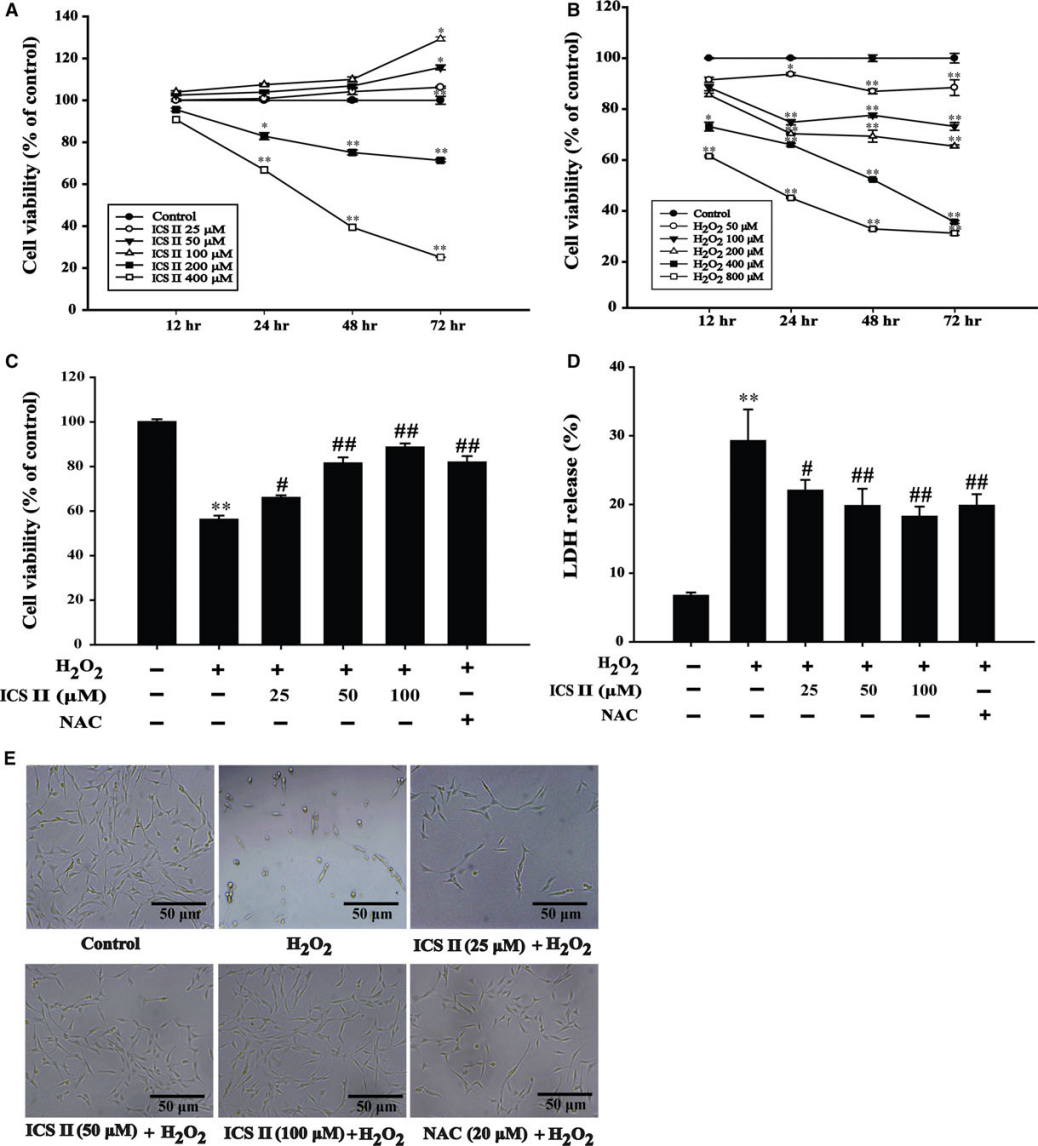
The effect of ICS II on H_2_O_2_ ‐cytotoxicity in PC12 cells. (**A**) PC12 cells were treated with different concentrations of ICS II for 12–72 hrs, and cell viability was determined by the MTT assay. (**B**) PC12 cells were treated with varying concentrations of H_2_O_2_ (50, 100, 200, 400 and 800 μM) for 12, 24, 48 and 72 hrs. (**C**) PC12 cells were pre‐treated with different concentrations of ICS II (25, 50,100 μM) for 1 hr. Thereafter, cells were treated with 400 μM H_2_O_2_ for 48 hrs, and cell viability was determined by MTT assay. (**D**) LDH release from PC12 cells was determined by an LDH release assay. (**E**) The effect of ICS II on H_2_O_2_‐induced morphological changes in PC12 cells. Data are presented as mean ± S.D. of three independent experiments. *P < 0.05, **P < 0.01 *versus* untreated control cells; ^#^ *P* < 0.05, ^##^ *P* < 0.01 *versus* H_2_O_2_‐treated cells.

This study evaluated in a first step the effect of ICS II on neuron cell viability and proliferation measured by the MTT test. ICS II (25–100 μM) had no significant effect on PC12 cells, whereas higher ICS II (200–400 μM) significantly inhibited cell growth compared to the untreated group within 72 hrs [*F*(5,12) = 9.571, *P* = 0.001; *F*(5,12) = 91.425, *P* < 0.001; *F*(5,12) = 82.333, *P* < 0.001; *F*(5,12) = 54.177, *P* < 0.001 respectively]. Therefore, ICS II at concentrations of 25–100 μM was used in the following experiments, considering that ICS II (25–100 μM) did not induce significant cytotoxity, as demonstrated by MTT measure (Fig. 2A).

Thereafter, we evaluated the effect of H_2_O_2_ (50–800 μM) on PC12 cells. H_2_O_2_ decreased cell viability in a dose‐ and time‐dependent manner [*F*(5,17) = 18.795, *P* < 0.001; *F*(5,17) = 270.141, *P* < 0.001; *F*(5,17) = 205.303, *P* < 0.001; *F*(5,17) = 300.517, *P* < 0.001 respectively]. Considering that 48 hrs exposure to 400 μM H_2_O_2_ incubation significantly decreased the viability of PC12 cells to approximately 50%, such dose and incubation time were used for the following experiments (Fig. 2B).

We investigated thereafter whether ICS II can protect PC12 cells from H_2_O_2_‐induced cell death. Pre‐treatment of PC12 cells with 25–100 μM ICS II significantly attenuated H_2_O_2_‐induced decrease of metabolically active cells in a dose‐dependent manner [*F*(5,17) = 60.607, *P <* 0.001] (Fig. 2C). In parallel, after exposure to 400 μM H_2_O_2_ for 48 hrs, LDH release was significantly higher in H_2_O_2_‐treated cells than in control cells, indicating that H_2_O_2_ was toxic to PC12 cells. In contrast, the LDH release was decreased by pre‐treatment with ICS II in a dose‐dependent manner [*F*(5,17) = 74.929, *P <* 0.001] (Fig. 2D). Moreover, this protective effect of ICS II was also confirmed by morphologic observations of cells. In the case of H_2_O_2_‐treated cells, most cells lost normal morphological characteristics: the shrinkage and floatation markedly differed from the characteristics of the adherent cells in the control case. After pre‐treatment with ICS II or NAC, the majority of H_2_O_2_‐treated cells grew with normal morphology and growth rate. The protective effects of ICS II were equal or higher than those exerted by an equivalent dose of NAC (Fig. 2E).

### ICS II attenuated H_2_O_2_‐induced intracellular and mitochondrial ROS generation and restored MPP

**Figure 3:**
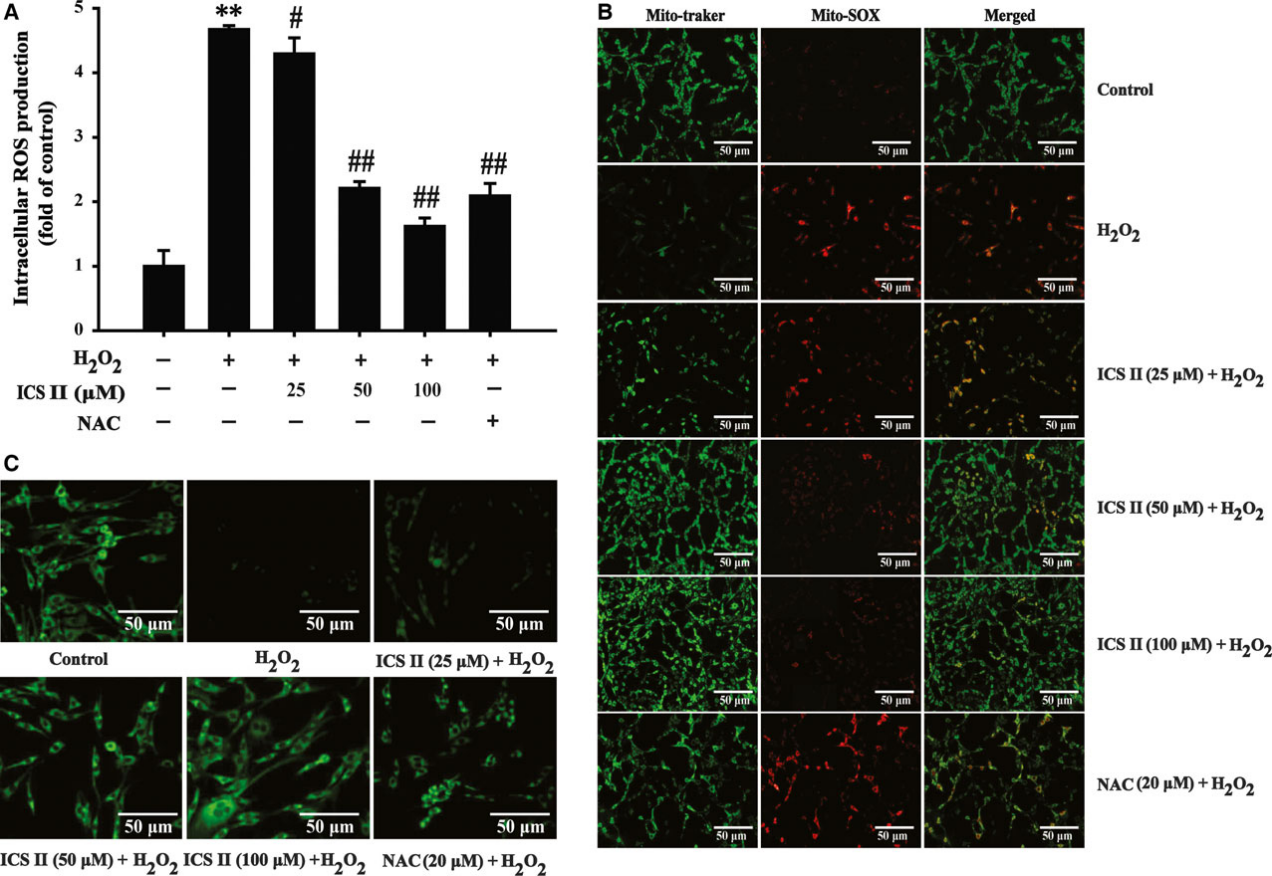
The effect of ICS II on intracellular and mitochondrial ROS level and on MMP change. After pre‐treatment of PC12 with ICS II for 1 hr, cells were treated with 400 μM H_2_O_2_ for 48 hrs. (**A**) intracellular ROS level determined with DCFH‐DA dye. (**B**) mitochondrial ROS level determined with MitoSOX Red dye. (**C**) MMP determined with rhodamine 123 dye. The result shown in A is presented as the mean ± S.D. of three independent experiments. **P < 0.01 *versus* untreated control cells; ^#^ *P* < 0.05, ^##^ *P* < 0.01 *versus* H_2_O_2_‐treated cells.

Because the H_2_O_2_‐induced cytotoxicity is known to be mediated mainly by oxidative stress, we used DCFH‐DA fluorescence to investigate whether ICS II affected intracellular ROS formation triggered by H_2_O_2_ exposure of cells. After cells were exposed to H_2_O_2_, the levels of intracellular ROS increased significantly within 48 hrs. Pre‐treatment with ICS II (25–100 μM) suppressed H_2_O_2_‐triggered ROS burst [*F*(5,17) = 52.145, *P <* 0.001] (Fig. 3A). Furthermore, to confirm the generation of oxidative stress in mitochondria during H_2_O_2_ treatment, PC12 cells were loaded with MitoSOX, a cationic probe that distributes to the mitochondrial matrix and specifically detects superoxide anion. A significant increase in mitochondrial superoxide generation was observed in H_2_O_2_‐treated cells for 48 hrs as compared to control. This effect was significantly attenuated by ICS II pre‐treatment (Fig. 3B).

Mitochondria are the major source of superoxide anion and other ROS which could determine the fate of cells through regulating many signalling pathways 24. MMP reflects the function of mitochondria. Cells exposed to H_2_O_2_ for 48 hrs, led to the decrease in rhodamine 123 fluorescence; whereas this decrease in rhodamine 123 fluorescence was abolished by pre‐treatment with ICS II or NAC (Fig. 3C). The results suggested that H_2_O_2_ impairs mitochondria *via* triggering MMP, while pre‐treatment with ICS II dramatically abrogated the decrease of MMP compared to H_2_O_2_ alone.

### ICS II ameliorated H_2_O_2_‐induced PDE 5 level and increased nitric oxide and cGMP level

**Figure 4:**
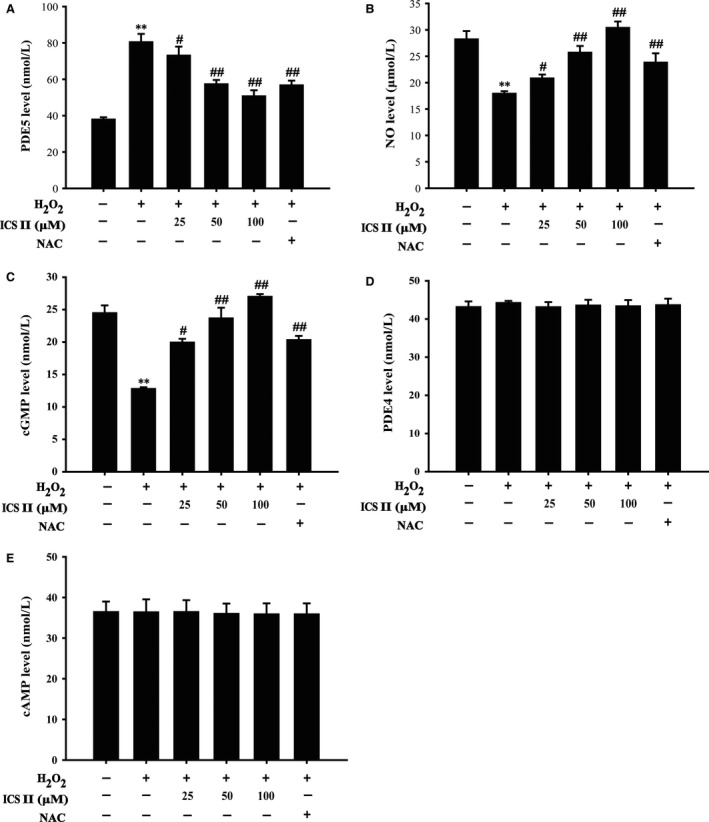
Effects of ICS II on PDE5, nitric oxide, PDE4, cGMP and cAMP level. Cells were pretreated with or without ICS II for 1 hr and then incubated with 400 μM H_2_O_2_ for 48 hrs, PDE5, nitric oxide, PDE4, cGMP and cAMP levels were examined by ELISA. (**A**) PDE5 (**B**) Nitric oxide. (**C**) cGMP. (**D**) PDE4. (**E**) cAMP. Data are presented as the mean ± S.D. of three independent experiments. **P < 0.01 *versus* untreated control cells; ^#^ *P* < 0.05, ^##^ *P* < 0.01 *versus* H_2_O_2_‐treated cells.

The level of nitric oxide and the protein level of PDE4, PDE5, cAMP and cGMP were assayed by ELISA. Treatment with 400 μM H_2_O_2_ for 48 hrs increased PDE5 level and decreased nitric oxide and cGMP level compared with control. Although pre‐treatment with ICS II decreased the level of PDE5 [*F*(5,17) = 75.312, *P <* 0.001] and increased the level of nitric oxide and cGMP [*F*(5,17) = 74.086, *P <* 0.001; *F*(5,17) = 20.347, *P <* 0.001 respectively] (Fig. 4A–C), but, ICS II had no effect on the level of PDE4 and cAMP [*F*(5,17) = 0.017, *P =* 1.000; *F*(5,17) = 0.031, *P =* 0.999 respectively] (Fig. 4D–E).

### ICS II protected PC12 cells against H_2_O_2_‐induced cell death by regulating GSK‐3β

**Figure 5:**
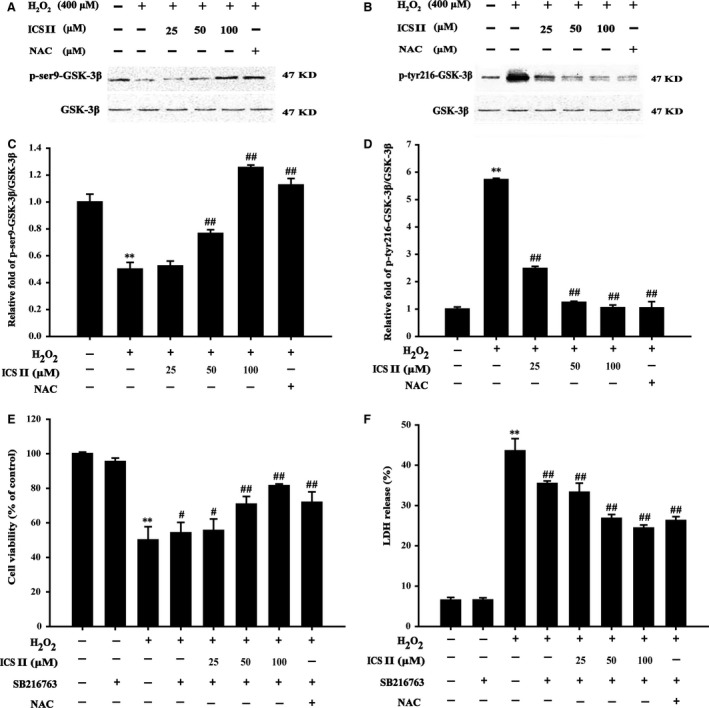
Effects of ICS II on the expression of GSK‐3β protein. (**A**) A representative Western blot is shown for ser9‐GSK‐3β protein. (**B**) A representative Western blot is shown for tyr216‐GSK‐3β protein. (**C**) Quantitation of phosphorylation of ser9‐GSK‐3β protein. (**D**) Quantitation of phosphorylation of tyr216‐GSK‐3β protein. The relative optical density was normalized to total GSK‐3β. (**E**) The effect of SB216763 and ICS II on the viability of PC12 cells evaluated by the MTT test. (**F**) The effect of SB216763 and ICS II on LDH release in PC12 cells. Data are presented as the mean ± S.D. of three independent experiments. **P < 0.01 *versus* untreated control cells; ^#^ *P* < 0.05, ^##^ *P* < 0.01 *versus* H_2_O_2_‐treated cells.

To determine whether GSK‐3β is involved in the signalling pathway of H_2_O_2_‐induced cell death, GSK‐3β expression was examined. After H_2_O_2_ treatment for 48 hrs, the protein levels of phospho‐tyr216‐GSK‐3β and phospho‐ser9‐GSK‐3β expressed by PC12 cells were increased and decreased respectively. ICS II pre‐treatment reduced the phosphorylation of GSK‐3β at tyr216 and increased the phosphorylation of GSK‐3β at ser9 [*F*(5,17) = 155.683, *P <* 0.001; *F*(5,17) = 141.022, *P <* 0.001 respectively], indicating that the autophagic process was inhibited (Fig. 5A–D). To determine the role of GSK‐3β, a GSK‐3β inhibitor SB216763 was used to modulate H_2_O_2_‐induced cytotoxicity. Cell viability assessed by the MTT test was increased in the presence of SB216763 when compared with H_2_O_2_ alone [*F*(7,16) = 50.058, *P <* 0.001] (Fig. 5E). Analysis of LDH release confirmed the suppression of H_2_O_2_‐induced cytotoxicity by SB216763, suggesting a role of GSK‐3β activation in H_2_O_2_‐induced cell death [*F*(7,16) = 233.197, *P <* 0.001] (Fig. 5F).

### ICS II attenuated H_2_O_2_‐induced autophagy in PC12 cells

**Figure 6:**
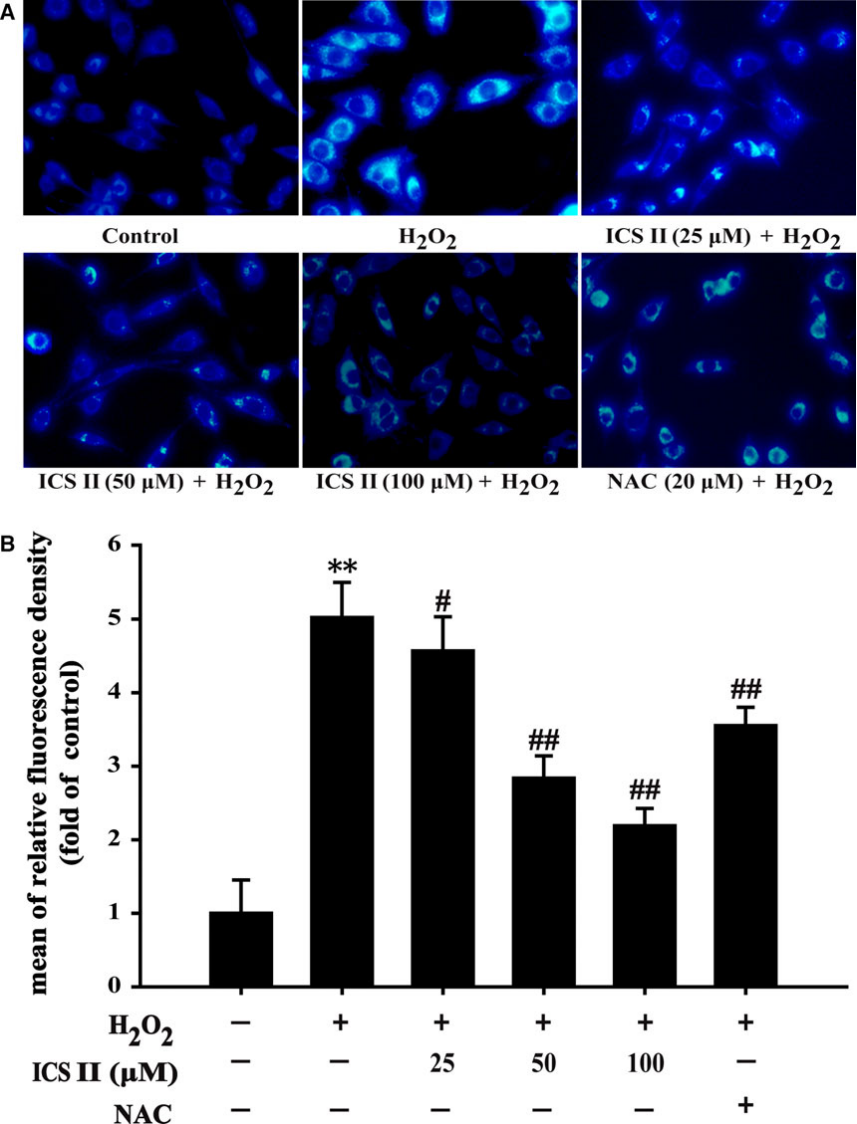
The effect of ICS II on H_2_O_2_‐induced autophagosomes in PC12 cells. (**A**) Autophagic vacuole formation was observed by fluorescence microscope. (**B**) The mean fluorescence intensity was measured by Image Pro Plus software. The result shown in B is presented as the mean ± S.D. of three independent experiments. **P < 0.01 *versus* untreated control cells; ^#^ *P* < 0.05, ^##^ *P* < 0.01 *versus* H_2_O_2_‐treated cells.

**Figure 7:**
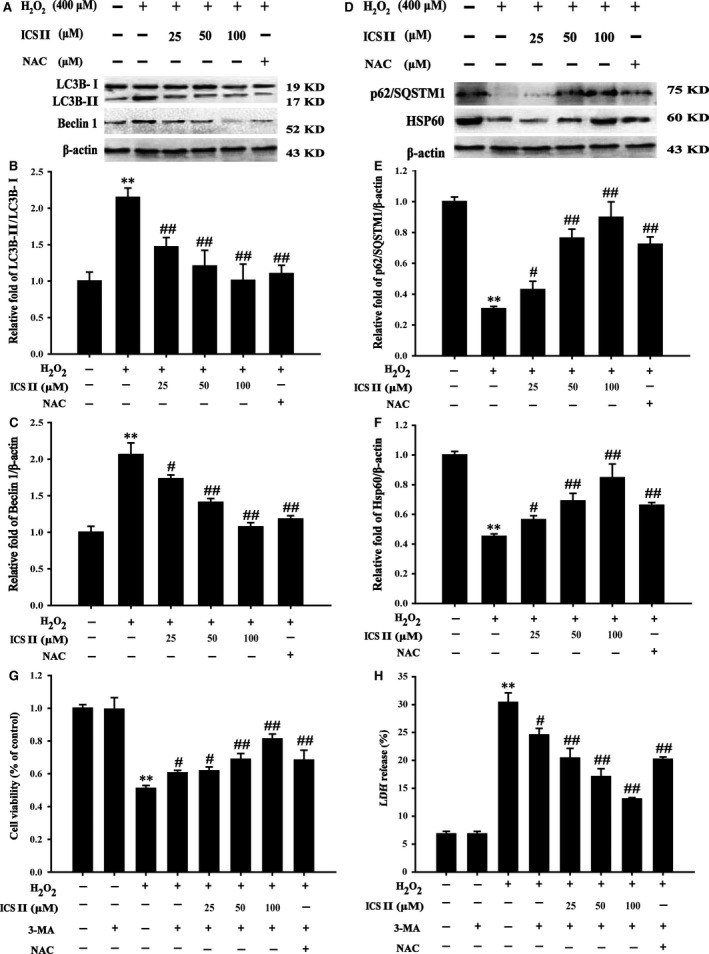
The role of ICS II in H_2_O_2_‐induced autophagy. (**A**) Representative Western blot image of LC3‐I, LC3‐II and Beclin 1. (**B**) Quantitation of LC3‐II/LC3‐I. (**C**) Quantitation of Beclin 1. (**D**) Representative Western blot image of p62 and HSP60. (**E**) Quantitation of p62/SQSTM1 protein. (**F**) Quantitation of HSP60 protein. H_2_O_2_‐induced autophagy in response to 3‐MA. (**G**) Cell viability analysed using the MTT assay. (**H**) Cytotoxicity analysed by the LDH release assay. Data are presented as the mean ± S.D. of three independent experiments. **P < 0.01 *versus* untreated control cells; ^#^ *P* < 0.05, ^##^ *P* < 0.01 *versus* H_2_O_2_‐treated cells.

To determine whether ICS II modulated H_2_O_2_‐induced autophagy, autophagic flux was determined. The suppressive effect of ICS II on autophagy was highlighted by the staining of autophagic vacuoles with MDC, a lysosomotropic compound known to label acidic endosomes, lysosomes and autophagosomes. Pre‐treatment with ICS II dramatically decreased the number of MDC‐labelled vesicles, suggesting that ICS II could reduce the formation of acidic vesicular organelles, a characteristic of autophagy [*F*(5,12) = 52.980, *P <* 0.001] (Fig. 6). ICS II pre‐treatment notably decreased the LC3‐II/LC3‐I ratio. The expression of Beclin‐1, an upstream promoter of the autophagy pathway, was also markedly attenuated by ICS II [*F*(5,12) = 35.181, *P <* 0.001; *F*(5,12) = 24.359, *P <* 0.001 respectively] (Fig. 7A–C). Moreover, ICS II pre‐treatment restored expression of p62/Sequestome 1 protein (p62/SQSTM1), which is directly degraded by and therefore serves as a marker of autophagic flux [*F*(5,12) = 145.888, *P <* 0.001]. In addition, HSP60 protein, which is an essential mitochondrial chaperone and promotes the folding of many proteins imported into the mitochondrial matrix was also increased by pre‐treatment with ICS II [*F*(5,12) = 64.671, *P <* 0.001] (Fig. 7D–F). Pharmacological inhibition of autophagy by 3‐MA resulted in an increase in cell viability compared with exposure to H_2_O_2_ alone [*F*(7,16) = 82.881, *P <* 0.001] (Fig. 7G). Parallel analysis of LDH release confirmed the suppression of H_2_O_2_‐induced cytotoxicity by 3‐MA, suggesting a role of autophagy in H_2_O_2_‐induced cell death [*F*(7,16) = 56.263, *P <* 0.001] (Fig. 7H).

## Discussion

Oxidative stress and altered redox signalling are responsible for various redox diseases. In the central nervous system overproduction of H_2_O_2_ induces cellular oxidative stress and tissue damage, and aberrant release of H_2_O_2_ is thought to be the major initiator of other ROS. Therefore, H_2_O_2_ is often used as a toxicant *in vitro* models to mimic oxidative stress‐induced neuronal injury 25. Our results demonstrated that ICS II can protect against oxidative stress‐induced PC12 cells injury. In addition, nitric oxide/cGMP signalling was correlated with the protective effect of ICSII on PC12 cells, which is in accordance with the beneficial effect of ICS II on neurogenic erectile dysfunction by the nitric oxide/cGMP pathway in the peripheral nervous system. Interestingly, ICS II had no effect on PDE4 activity nor cAMP, which was consistent with the theory that PDE 4 mainly hydrolyzes cAMP, and PDE5 is 100‐fold more selective for cGMP than cAMP 12. Thus, our data indicated that ICS II is a selective PDE5 inhibitor.

Because most of the neuronal energy transducing pathways occur in mitochondria, it is reasonable to speculate that mitochondrial H_2_O_2_ participates in the regulation of redox‐sensitive signalling 26. The present study demonstrated that ICS II had beneficial effect on oxidative stress and excessive mitochondrial ROS generation that may induce mitochondrial dysfunction. Previous studies reported that H_2_O_2_ is the key trigger of elevated PDE5 activity and mitochondrial matrix oxidative signals in a cGMP‐dependent protein kinase‐dependent manner 27. Our findings confirmed that ICS II protected against H_2_O_2_‐induced neuron damage, at least in part by inhibiting PDE5, reducing ROS levels and restoring mitochondrial function.

ROS are acting upstream of GSK‐3β, and determine the translocation of GSK‐3β into the mitochondria. Activated GSK‐3β in mitochondria then binds to and phosphorylates the components of the mitochondrial membrane pore, and thereby inducing MMP transition 28. Thus, GSK‐3β acts as a pivotal kinase for the regulation of MMP transition, which is crucial for cell viability. The results demonstrated that exposure of PC12 cell to H_2_O_2_ could induce GSK‐3β activation by regulating site‐specific phosphorylation and that ICS II at least protects against ROS‐mediated mitochondrial dysfunction through inactivation of GSK‐3β. ICS II as a PDE5 inhibitor inactivated GSK‐3β‐related nitric oxide/cGMP signalling pathway, which is in agreement with the theory that PDE5 inhibitors can inactivate GSK‐3β through cGMP‐dependent pathway.

Autophagy, known as programmed cell death type II (autophagic death), serves as the housekeeping function under normal conditions. However, excessive autophagy can result in cell death, and accumulating evidence suggests the complex crosstalk between mitochondria, autophagy and ROS 29. LC3 is located on the membrane surfaces of pre‐autophagic and autophagic vacuoles. LC3‐II reflects autophagy activity to some extent and it is a common membrane marker for autophagic vacuoles. Beclin 1 participates in the formation of autophagosomes and plays an important role in cell growth by regulating autophagic activity 30. p62/SQSTM1 is a multifunctional ubiquitinated protein coupled to LC3, which is involved in the formation of autophagosomes as a regulatory factor and is degraded in the middle or late‐phase of autophagy. Therefore, the total expression level of intracellular p62/SQSTM1 was negatively correlated with autophagic activity 31. The findings in this study showed that H_2_O_2_‐induced mitochondrial ROS accumulation was accompanied by autophagosome accumulation, increased the ratio of LC3‐II/LC3‐I and Beclin 1 levels, and decreased p62/SQSTM1 expression, hence indicating H_2_O_2_ activated autophagic flux. ICS II repressed H_2_O_2_‐induced autophagic flux. Moreover, we found ICS II increased the level of HSP60, a protein that is predominantly found in the mitochondria matrix and participates in the maintenance of mitochondrial integrity and inhibition of autophagosome formation 32. Furthermore, the autophagy inhibitor 3‐MA suppressed oxidative stress‐induced cell death, suggesting that autophagy is a consequence of the increased ROS and there exists a cross‐talk between oxidative stress, mitochondria, and autophagy.

**Figure 8:**
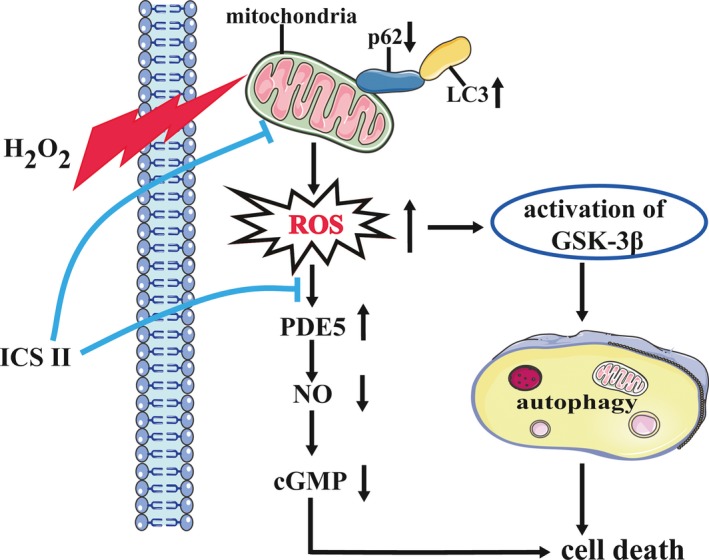
Schematic presentation of a proposed mechanism for the protective role of ICS II against H_2_O_2_‐induced oxidative stress in PC12 cells. H_2_O_2_ is thought to be the major precursor of ROS, and accumulated intracellular ROS trigger mitochondrial dysfunction, activation of GSK‐3β, and autophagic cell death. ICS II, a PDE5 inhibitor, attenuates ROS production and autophagy induced by H_2_O_2_ *via* ROS/GSK‐3β/mitochondrial signalling pathways.

In summary, this study demonstrates that the PDE5 inhibitor ICS II attenuated H_2_O_2_‐induced mitochondrial dysfunction and autophagy at high H_2_O_2_ concentrations to which rat neuronal PC12 cells were experimentally exposed. Furthermore, we pointed out that the suppression of the ROS/GSK‐3β signalling pathway by ICS II significantly reduced H_2_O_2_‐induced intracellular ROS levels and autophagy (Fig. 8). Results of the present study demonstrated that ICS II is a promising PDE5 inhibitor that modulates intracellular oxidative activity and antioxidant response in rat neuronal PC12 cells, hence having therapeutic potential in redox‐mediated neurological diseases.

## Conflict of interest

The authors declare no conflict of interest.
